# Effect of the ati Gene Deletion on the Pathogenicity and Immunogenicity of the Vaccinia Virus

**DOI:** 10.32607/actanaturae.17872

**Published:** 2023

**Authors:** S. N. Yakubitskiy, A. A. Sergeev, K. A. Titova, I. S. Shulgina, E. V. Starostina, M. B. Borgoyakova, L. I. Karpenko, S. N. Shchelkunov

**Affiliations:** State Research Center of Virology and Biotechnology VECTOR, Rospotrebnadzor, Koltsovo, Novosibirsk region, 630559 Russian Federation

**Keywords:** orthopoxviruses, vaccinia virus, ati gene, intradermal injection, immunogenicity, protectivity

## Abstract

Among the nonvirion proteins of the vaccinia virus (VACV), a 94-kDa long protein is most abundantly present; the protein is a truncated form of the 150-kDa A-type inclusion (ATI) protein of the cowpox virus encoded by the *ati *gene. This VACV protein does not form intracellular ATIs, being as it is a major immunogen upon infection/immunization of humans or animals with the VACV. Antibodies specific to this protein are not virus-neutralizing. The present study focused on the effect of the production of this nonstructural major immunogenic VACV protein on the manifestation of pathogenicity and immunogenicity of the virus in the BALB/c mouse model of infection. In order to introduce a targeted deletion into the VACV LIVP genome, the recombinant integration/deletion plasmid pΔati was constructed and further used to generate the recombinant virus LIVPΔati. The pathogenicity of the VACV LIVP and LIVPΔati strains was studied in 3-week-old mice. The mice were intranasally infected with the viruses at a dose of 107 pfu; 50% of the animals infected with the parent LIVP strain died, while infection with the LIVPΔati strain led to the death of only 20% of the mice. Intradermal vaccination of mice aged 6– weeks with the LIVPΔati virus statistically significantly increased the production of VACV-specific IgG, compared to that after intradermal vaccination with VACV LIVP. Meanwhile, no differences were noted in the cell-mediated immune response to the vaccination of mice with VACV LIVP or LIVPΔati, which was assessed by ELISpot according to the number of splenocytes producing IFN-γ in response to stimulation with virus-specific peptides. Intranasal infection of mice with lethal doses of the cowpox virus or the ectromelia virus on day 60 post-immunization with the studied VACV variants demonstrated that the mutant LIVPΔati elicits a stronger protective response compared to the parent LIVP.

## INTRODUCTION

The vaccinia virus (VACV) belongs to the genus* Orthopoxvirus *(family Poxviridae), which also comprises such virus species as the variola virus (VARV), monkeypox virus (MPXV), cowpox virus (CPXV), camelpox virus (CMLV), and others [1, 2]. Orthopoxviruses are the largest DNA-containing mammalian viruses whose entire life cycle occurs in the cytoplasm of infected cells. The members of this genus are morphologically indiscernible in terms of virion structure and antigenically close to each other; therefore, infection with one orthopoxvirus species affords immunity against other members of this genus [3]. For this very reason, smallpox has been eradicated using the live vaccine based on different VACV stains [1, 4].

Like for other orthopoxviruses, there exist two infectious forms of VACV. Intracellular mature virions (IMVs) make up the overwhelming majority of virus progeny, while a much smaller portion is represented by extracellular enveloped virions (EEVs) [5, 6].

The so-called LS antigen (an antigen complex consisting of the thermolabile (L) and thermostable (S) antigenic components), against which antibodies are intensely produced, was discovered in early studies focusing on the immune response elicited by intradermally (i.d.) infecting rabbits with VACV [7]. The highly immunogenic LS antigen, which is a nonvirion protein and is abundantly present, is isolated from the extracts of infected animal tissue. The antibodies produced after the animals are immunized with LS antigen exhibit no virus-neutralizing activity but react to clinical samples collected from patients with smallpox and monkeypox in the complement binding and gel immunoprecipitation assays [2].

**Fig. 1:**
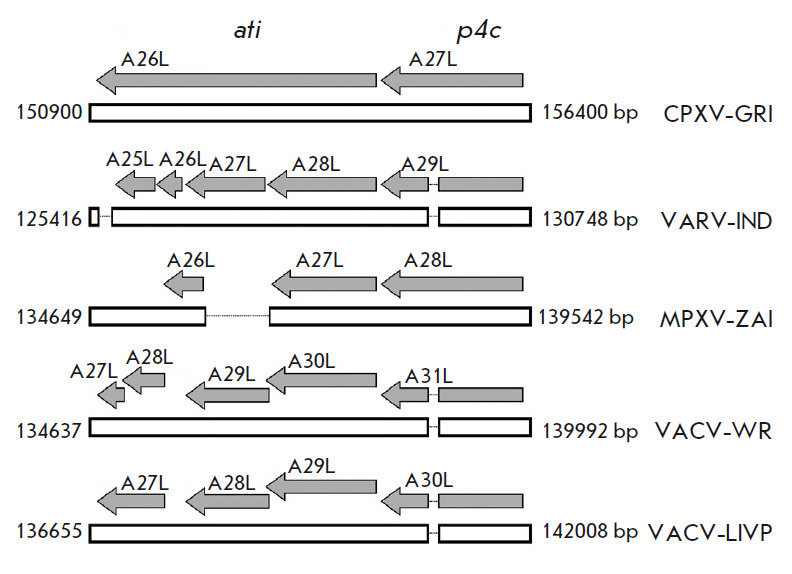
Comparison of the potential orthopoxvirus open reading frames (ORFs) within the *ati *(ORF A26L) and* p4c *((A27L) CPXV-GRI) genes. Gray arrows indicate the size and direction of the respective ORFs; their names are provided above these arrows for the CPXV-GRI, VARV-IND, MPXV-ZAI, VACV-WR [2], and VACV-LIVP viruses. Numbers to the right and left of white boxes denote the positions of the respective regions of the viral genomes. Thin lines indicate deletions in the viral genomes with respect to the CPXV-GRI DNA sequence. The truncated form of the ATI protein is encoded by ORF A29L VACV-LIVP and the respective ORFs of other viruses

It was demonstrated later that the 94-kDa LS antigen of VACV is the truncated form of the protein that forms intracellular A-type inclusion bodies (ATIs) in CPXV infected cell. The ATI protein of CPXV is 150 kDa in size [8], is encountered in large quantities in infected cells during the later stage of the virus replication cycle (up to 4% of total cell protein), and is aggregated to yield gel-like bodies that may incorporate mature virions in their cytoplasm [9, 10]. C-terminally truncated forms of this protein 92–96 kDa in size are synthesized in large quantities by VARV, MPXV, CMLV, and VACV, without forming ATIs (*Fig. 1*).

It was demonstrated that immunization of laboratory animals or human volunteers with VACV results in the production of antibodies against a broad range of virion proteins, including the highly immunogenic nonvirion ATI-like protein [11]. The T-cell-mediated immune response to a VACV infection is mainly induced against early nonvirion proteins [6]. The only exception is the truncated ATI protein synthesized during the late stage of the virus replication cycle; nevertheless, a strong immune response is elicited to it [12]. A plausible reason is that the truncated *ati* gene (*A29L* in the case of VACV-LIVP, see *Fig. 1*) is one of the most intensely expressed VACV genes, and that the protein encoded by it is produced in the largest quantities among nonvirion proteins [9].

The *ati *gene is not among the essential genes for the VACV, since natural variants of the virus with this gene deleted have been discovered [13, 14]. It was also demonstrated for CPXV that *ati *gene deletion affects viral replication ability neither *in vitro *nor *in vivo *[15, 16]. However, the effect of *ati *gene deletion on the immunogenic properties of VACV still remains unclear.

This study focused on the impact of the deletion of the *ati *gene, which encodes the major immunogenic protein of VACV (antibodies specific to it exhibit no virus-neutralizing activity), on the pathogenicity and immunogenicity of the virus.

## MATERIALS AND METHODS

**Bacteria, viruses, and cell culture**

The *Escherichia coli *XL2-Blue strain and LIVP VACV cl. 14 obtained previously by finite dilution in agarose gel [17], as well as the cowpox virus (CPXV) strain GRI-90 and the ectromelia virus (ECTV) strain K-1, obtained from the virus collection, were used in this study. The passaged African green monkey kidney cell culture CV-1 was procured from the Cell Culture Collection of the State Research Center of Virology and Biotechnology VECTOR, Rospotrebnadzor. The viruses were grown and titrated using a monolayer CV-1 culture according to the procedure described in [18].

**The Animals**

BALB/c inbred mice procured from the husbandry of the Institute of Cytology and Genetics, Siberian Branch of the Russian Academy of Sciences (Novosibirsk, Russia) were used for the study. The experimental animals were fed the standard diet with a sufficient amount of water, in compliance with the veterinary legislation and requirements for humane care and use of laboratory animals (State Standard GOST 33216-2014 “Guidelines for Accommodation and Care of Animals. Species-specific Provisions for Laboratory Rodents and Rabbits”). The studies and manipulations involving animals were approved by the Bioethics Committee of the State Research Center of Virology and Biotechnology VECTOR, Rospotrebnadzor (Protocol No. 02-06.2022 dated June 28, 2022).

**Pathogenicity assessment of the VACV strains**

Three-week-old BALB/c mice (10 animals per group) were used in the study focusing on the pathogenicity of the VACV LIVP and LIVPΔati viruses (for intranasal (i.n.) infection). After inhalation anesthesia with diethyl ether, the mice received either a virus-containing fluid (50 μL, 107 plaque-forming units (pfu)) or saline into the nasal cavity. The animals were followed up for 22 days; clinical manifestations of infection and animal death were documented.

The following scoring system for disease symptoms was used: 0 – no disease signs; 1 – slightly unkempt hair coat; 3 – significantly unkempt hair coat, as well as back-arching or conjunctivitis; 4 – hard breathing or immobility; and 5 – death.

Each mouse was weighed individually every two days. The arithmetic mean body weights of the mice in each group at a given time point were calculated and expressed as a percentage of the initial weight.

**Immunization of mice and sample collection for analyses**

BALB/c mice aged 6–7 weeks were immunized with the LIVP and LIVPΔati VACV strains by making an intradermal (i.d.) injection into the dorsal side of the tail (~ 1 cm from the tail base), according to the procedure described earlier [19] using a virus dose of 105 pfu/20 μL/mouse. The mice that had received saline were used as negative controls.

The humoral immune response in the mice was analyzed on days 7, 14, 21, 28, 42, and 56 post immunization (dpi). At each time point specified above, six mice per group were taken for analysis. Mouse blood samples were intravitally collected from the retro-orbital venous sinus using a 23G needle. Blood sampling from the retro-orbital sinus does not cause visual organ injury. Blood sampling is a short-lasting but painful procedure; however, no analgesia was used, as it has been demonstrated that the known analgesic or anesthetic techniques may affect the immunological parameters of the animal’s blood.

Sera were obtained from individual animal blood samples by precipitation of blood cells via centrifugation at a relative centrifugal force of 1,000 *g *during 10 min. The sera were exposed at 56°C during 30 min and stored at –20°C.

On 7, 14, and 21 dpi, after blood collection, the mice were euthanized by cervical dislocation; spleens were isolated from each out of six mice in the study groups under sterile conditions.

**Splenocyte separation**

Splenocytes were separated by passing individual spleens through a cell strainer (BD Falcon™, USA) using a syringe plunger. After red blood cells had been removed using a ACK lysing buffer (Thermo Fisher Scientific, USA), the splenocytes were washed and resuspended in a RPMI-1640 medium supplemented with 10% fetal bovine serum and gentamicin (50 μg/mL).

**Quantification of IFN-γ-producing cells by ELISpot**

The intensity of the T-cell-mediated immunity in the vaccinated mice was quantified by ELISpot according to the number of splenocytes producing IFN-γ. The assay was carried out using MABTECH kits (Sweden) in accordance with the manufacturer’s instruction. Cells were stimulated using a mixture of VACV-specific peptides SPYAAGYDL, SPGAAGYDL, VGPSNSPTF, KYGRLFNEI, GFIRSLQTI, and KYMWCYSQV immunodominant for BALB/c mice (20 μg/mL of each peptide) [20, 21]. The counts of IFN-γ-producing cells were determined using an ELISpot reader (Carl Zeiss, Germany).

**Enzyme-linked immunosorbent assay of murine sera**

Enzyme-linked immunosorbent assay (ELISA) of mouse sera was carried out according to the procedure described in [18]. A preparation of LIVP VACV virions purified by sucrose cushion centrifugation was used as an antigen. All the analyzed mouse serum samples were titrated using a series of twofold dilutions (from 1 : 100 to 1 : 12,800). Titration was repeated the next day when conducting ELISA. The IgM and IgG titers were determined using solutions containing peroxidase conjugates of anti-mouse IgM and anti-mouse IgG (Sigma, USA), respectively. The IgM and IgG titers were determined in each analyzed serum sample (individually for each repeat and then averaged). The geometric mean logarithms of inverse titer of VACV-specific IgG or IgM were calculated for the study groups, and 95% confidence intervals were determined.

**Assessment of protective efficacy in immunized mice**

On 60 dpi, the groups of animals immunized with VACV LIVP or LIVPΔati or the ones in the control group were i.n. infected with CPXV GRI-90 at a dose of 2.0 × 10^6^ pfu/50 μL/mouse (six animals per group) or ECTV K-1 at a dose of 2.2 × 10^3^ pfu/50 μL/mouse (six animals per group). The animals were followed up for 14 days; clinical manifestations of infection and deaths were documented. Each mouse was weighed every 2 days. The arithmetic mean body weights of the mice in each group at each time point were calculated and expressed as a percentage of the initial weight.

The data were obtained for the animal groups i.d. immunized with VACV LIVP or LIVPΔati, as well as the groups of non-immunized and non-infected mice (negative control) or mice infected with CPXV GRI-90 or ECTV K-1 (positive control).

**Statistical analysis**

Statistical analysis and data comparison were performed using the standard methods, employing the Statistica 13.0 software package (StatSoft Inc. 1984– 2001). *P* < 0.05 were considered statistically significant.

## RESULTS

**Construction of the LIVPΔati virus**

**Fig. 2:**
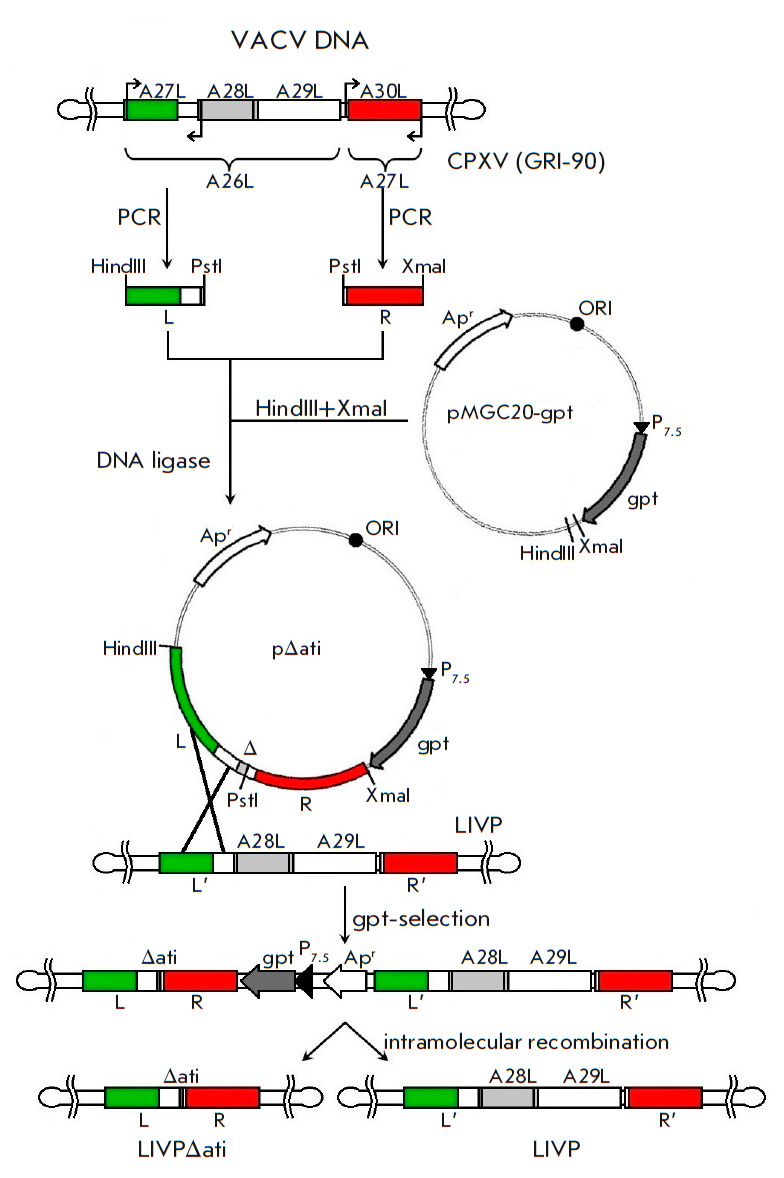
Scheme for constructing the plasmid pΔati and VACV LIVPΔati (see explanation in the text). L and R – left and right flanking *ati *gene regions

Targeted deletion in the VACV LIVP genome (GenBank: KX781953.1) was performed in accordance with the scheme shown in *Fig. 2*. At the first stage of constructing the recombinant integrative/deletion plasmid pΔati, we calculated and synthesized oligonucleotide primers for PCR and the amplification of flanking VACV LIVP DNA sequences adjacent to the left or right border of the viral genome region to be deleted (ORF A28L–A29L, position on the genome, 137618–140470 bp) using the Oligo software (version 3.3) (Borland International, USA) (*Fig. 1*, *Fig. 2*).

The left-border flanking fragment (L) was synthesized using the primer pair 5’-**AAGCTT**GTTTGGTAGTAGATACATATCAATATCATC- 3’ (**HindIII**) and 5’-**CTGCAG**GCTGACTCAATTGCATGAAGAT- 3’ (**PstI**); the right-border flanking fragment (R) was synthesized using the primers 5’-**CTGCAG**GGGTAATTATAAGATCGTAGATCTCC- 3’ (***Pst*I**) and 5’-**CCCGGG**ATGGCGAACATTATAAATTTATGG- 3’ (**XmaI**) and Platinum *Taq *DNA high-fidelity proofreading polymerase (Invitrogen, USA); DNA of LIVP VACV cl. 14 was used as the DNA template. The resulting target fragments L and R were purified using a QIAquick Gel Extraction Kit (QIAGEN, Netherlands) inserted into the vector HindIII–XmaI fragment pMGC20-gpt (*Fig. 2*) and cloned employing the transformation of competent *E. coli *cells (XL2-Blue strain) and ampicillin as a selective marker. Sequence correctness of the recombinant plasmid pΔati was confirmed by sequencing.

At the next stage of the study, the monolayer culture of CV-1 cells was infected with VACV LIVP and transfected with the recombinant plasmid pΔati, with simultaneous gpt selection of VACV recombinant according to the procedure described earlier [17]. Single crossover of the integrative plasmid and viral DNA gave rise to a recombinant viral genome carrying both the selective *gpt* gene and sequences represented by a viral genome segment carrying the target deletion and the same segment without the deletion (*Fig. 2*). This genetic construct carrying the long forward repeats R, R’ and L, L’ is unstable and can exist only under selective pressure [15, 16]. Withdrawal of selective pressure on the *gpt *gene and intramolecular recombination at R-R’ led to the formation of the recombinant virus LIVPΔati (*Fig. 2*). Clones of this viral variant were identified by PCR followed by sequencing of viral DNA.

**Assessment of the pathogenicity of the LIVP and LIVPΔati viruses upon intranasal infection of mice**

**Fig. 3:**
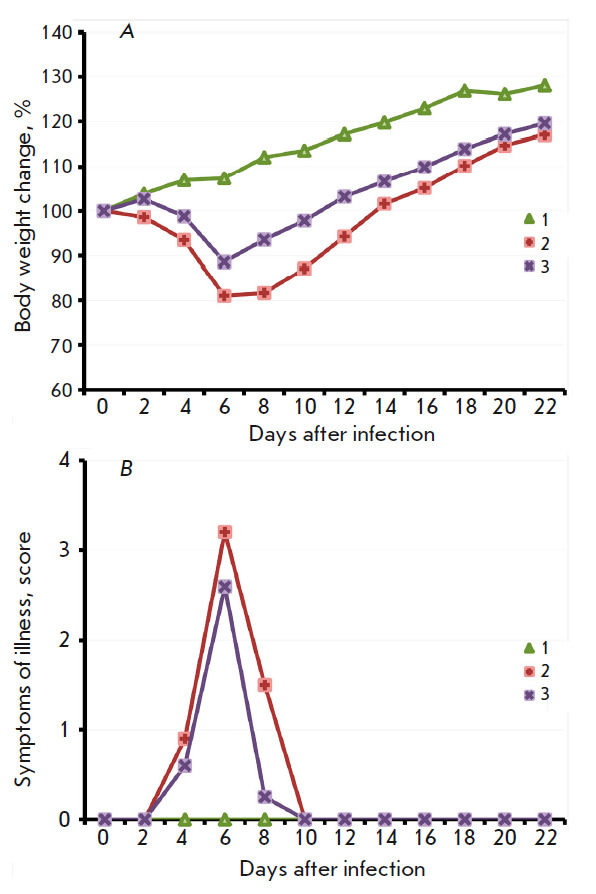
The dynamics of changes in mouse body weight (*A*) and clinical manifestations of the infection (*B*) after i.n. inoculation of the LIVP (*2*) or LIVPΔati (*3*) viruses at a dose of 107 pfu. Data for groups consisting of 10 animals infected with the respective viruses, as well as the control group (*1*), are presented

**Fig. 4:**
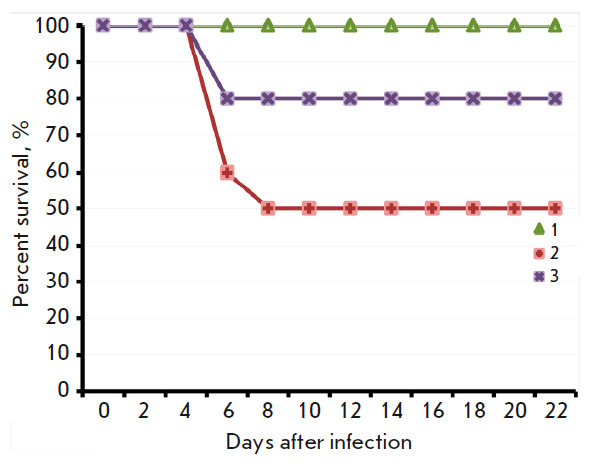
The dynamics of death of mice i.n. infected with the LIVP (*2*) or LIVPΔati (*3*) viruses at a dose of 107 pfu. Control group – non-infected animals (*1*)

The pathogenicities of the VACV LIVP and LIVPΔati strains were studied using 3-week-old BALB/c mice. The mice (10 animals per group) were i.n. infected with the viruses at a dose of 107 pfu. The animals were followed up during 22 days; each mouse was weighed; clinical manifestations of infection and deaths were documented. In mice infected with VACV LIVP, profound clinical manifestations of the infection were visible starting on day 4; the maximum was attained on day 6; and the animals had recovered after day 10 (*Fig. 3B*). Illness was accompanied by significant body weight reduction (*Fig. 3A*). Under the same conditions, the LIVPΔati virus caused less profound clinical manifestations of the infection (*Fig. 3B*) and less significant body weight loss compared to mice infected with LIVP (*Fig. 3A*), although the differences were statistically insignificant. Infection of mice with the LIVP strain led to the death of 50% of the animals, while only 20% of the mice infected with the LIVPΔati strain died (*Fig. 4*).

These findings are indicative of a reduced pathogenicity of the VACV LIVP with *ati* gene deletion (*Fig. 2*).

**Analysis of the development of a cellmediated immune response to vaccination of mice with VACV variants**

**Fig. 5:**
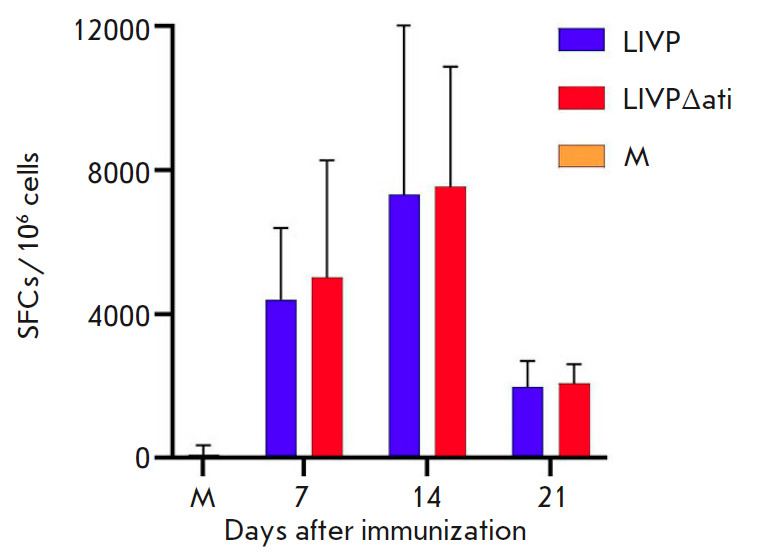
The results of ELISpot assay of the VACV-specific cell-mediated response in immunized BALB/c mice. SFCs – interferon-γ-producing cells; M – non-immunized mice

The intensity of the cell-mediated immune response in mice i.d. immunized with LIVP or LIVPΔati was determined on 7, 14, and 21 dpi by IFN-γ ELISpot, according to the number of splenocytes producing IFN-γ in response to stimulation with virus-specific peptides. Six animals per group were analyzed at each time point. The results shown in *Fig. 5* demonstrate that a high level of cell-mediated immune response was observed already on 7 dpi, peaking on 14 dpi and significantly dropping by 21 dpi. The dynamics and level of cell-mediated immune response coincided for both VACV strains.

**Comparison of the dynamics of emergence of a humoral immune response to the vaccination of mice with the LIVP and LIVPΔati viruses**

The levels of VACV-specific IgM and IgG in the sera of mice i.d. immunized with LIVP or LIVPΔati were determined by ELISA on 7, 14, 21, 28, 42, and 56 dpi.

**Fig. 6:**
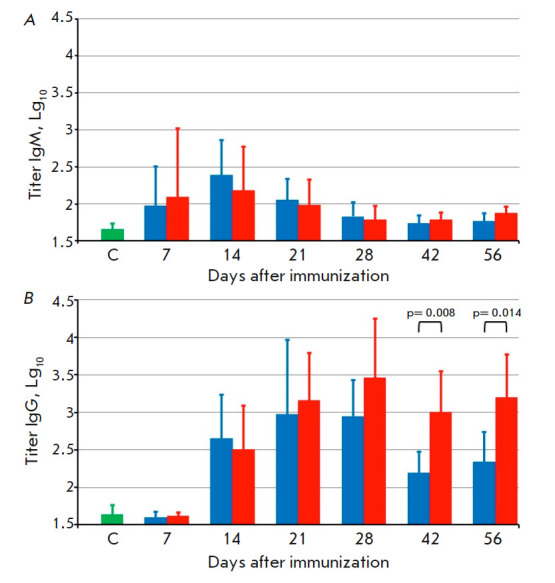
The titers of VACV-specific IgM (*A*) and IgG (*B*) in the sera of mice immunized with the LIVP (blue bars) or LIVPΔati (red bars) viruses. C – sera of mice that received saline

A relatively high IgM level was observed on 7 dpi; it reached its maximum on 14 dpi and then dropped. Both VACV strains did not differ in terms of the dynamics and level of production of virion-specific IgM (*Fig. 6A*).

An intense synthesis of VACV-specific IgG was observed on 14 dpi; the antibody level subsequently increased on 21–28 dpi and remained high throughout the entire follow-up period (up to 56 dpi, *Fig. 6B*) in mice immunized with LIVPΔati but declined in mice immunized with LIVP. In terms of the geometric mean of inverse IgG titers on 28, 42, and 56 dpi, LIVPΔati was noticeably superior to LIVP; these differences were statistically significant on 42 and 56 dpi (*Fig. 6B*).

**Assessment of the protective effect of immunization of mice with the VACV variants against challenge with lethal doses of heterologous orthopoxviruses**

On 60 dpi, mice i.d. immunized with VACV LIVP or LIVPΔati, as well as the control (non-immunized) mice, were i.n. infected with either CPXV (at a dose of 2.0 × 10^6^ pfu/mouse) or ECTV (at a dose of 2.2 × 10^3^ pfu/mouse) (six animals per group). During the 14-day follow-up period, we monitored clinical manifestations and deaths of mice. The criterion of VACV infection development according to body weight loss was used.

**Fig. 7:**
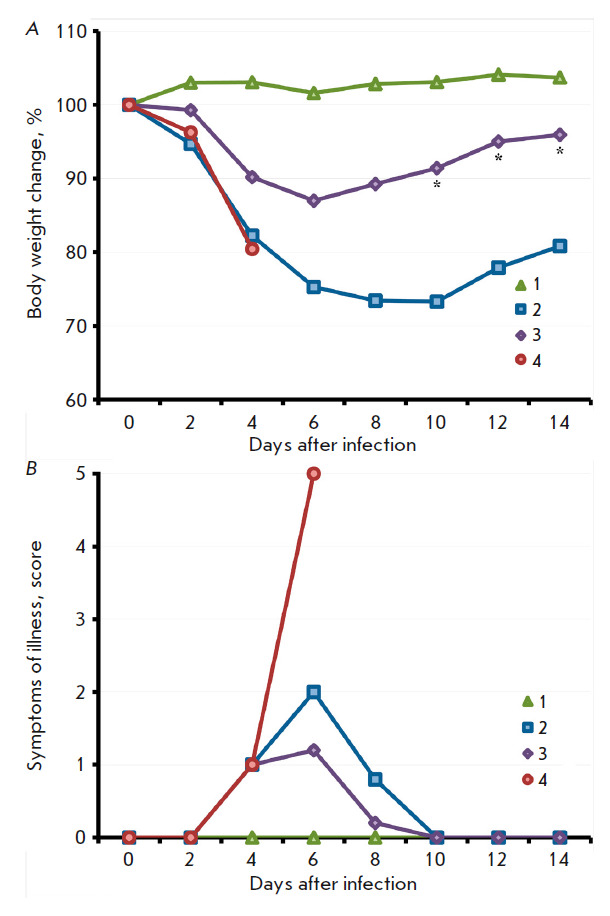
The dynamics of changes in body weight (*A*) and clinical manifestations of the infection (*B*) after i.n. infection of mice vaccinated with LIVP (*2*) or LIVPΔati (*3*) with CPXV-GRI on day 60 post-immunization. The data are presented for groups consisting of six animals. The controls were groups of non-vaccinated mice, both non-infected (*1*) and infected with CPXV-GRI (*4*). An asterisk shows the time points at which the mean body weights (expressed as a percentage of the initial weight) in the group of mice immunized with LIVPΔati differ statistically significantly from those in the group of mice immunized with LIVP. Comparison was performed using the Student’s t-test for independent samples

All the mice in the control group infected with CPXV died after 6 days, while the animals immunized with both VACV variants survived. Weight loss and clinical manifestations were less acute in mice vaccinated with LIVPΔati compared to those vaccinated with LIVP (*Fig. 7*). Differences in body weight loss between the groups of vaccinated mice were statistically significant on days 10–14 after infection with CPXV (*Fig. 7A*).

**Fig. 8:**
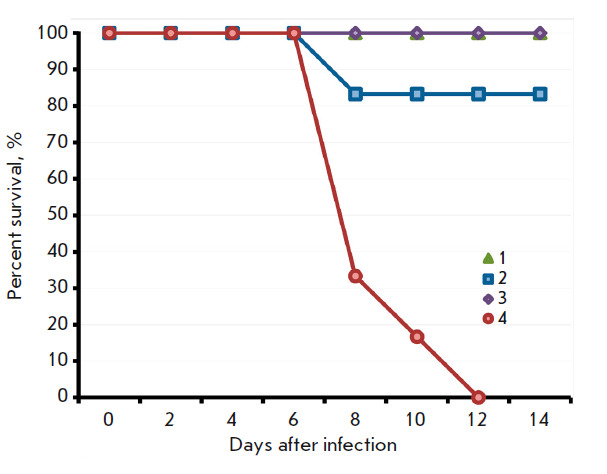
The dynamics of deaths of mice vaccinated with LIVP (*2*) or LIVPΔati (*3*) after i.n. infection with ECTV-K1 on day 60 post immunization. The data are presented for groups consisting of six animals. The controls were groups of non-vaccinated mice, both non-infected (*1*) and infected with CPXV-ECTV-K1 (*4*)

**Fig. 9:**
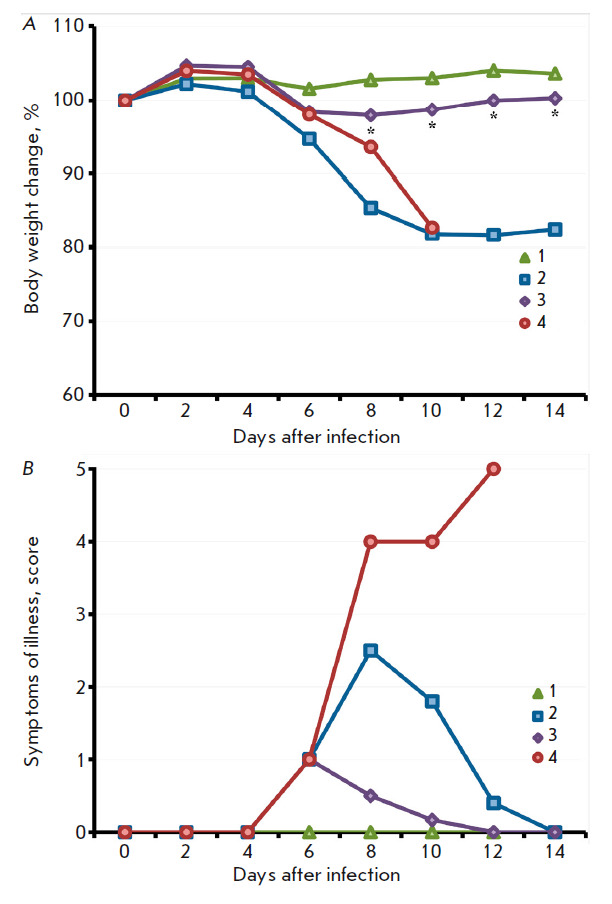
The dynamics of changes in body weight (*A*) and clinical manifestations of infection (*B*) after i.n. infection of mice vaccinated with LIVP (*2*) or LIVPΔati (*3*) with ECTV-K1 on day 60 post immunization. The data are presented for groups consisting of six animals. The controls were groups of non-vaccinated mice, both non-infected (*1*) and infected with ECTV-K1 (*4*). An asterisk shows time points at which the mean body weight (expressed as a percentage of the initial weight) in the group of mice immunized with LIVPΔati differs statistically significantly from that in the group of mice immunized with LIVP. Comparison was performed using the Student’s t-test for independent samples

Intergroup differences were more significant after the infection of immunized mice with the ECTV virus. All control mice died 12 days after infection with ECTV; 83% of the animals vaccinated with LIVP survived, while all the animals survived in the LIVPΔati group (*Fig. 8*). In the LIVPΔati group, manifestations of infection were very mild and were almost never accompanied by body weight loss (*Fig. 9*). Meanwhile, in mice vaccinated with LIVP, clinical signs of infection were observed on days 6–12 after infection with ECTV. The body weight of the animals had substantially dropped; statistically significant differences in this parameter were observed on days 8–14 after infection with ECTV compared to the group of mice vaccinated with LIVPΔati (*Fig. 9A*).

## DISCUSSION

The truncated form of the ATI protein (the 94-kDa protein that does not form intracellular ATIs but is a major immunogen upon infection/immunization of humans or animals with VACV) is synthesized most abundantly among nonvirion VACV proteins [11]. Meanwhile, antibodies specific to this protein do not exhibit any virus-neutralizing activity. The effect of the production of this nonstructural major immunogenic protein on the manifestation of pathogenicity and immunogenicity of VACV still needs research. Therefore, this work aimed to produce the VACV carrying a targeted deletion of the *ati *gene and investigate the properties of this virus in mice.

The previously characterized clonal variant of LIVP VACV [17] was used as the study object. In LIVP, the* ati *gene (*A26L* in CPXV-GRI) falls into three short potential open reading frames (ORFs); of those, the *A29L* gene encodes the major immunogenic protein A29 (94 kDa) (*Fig. 1*).

The integrative/deletion plasmid pΔati, and subsequently the LIVPΔati strain, were constructed according to the scheme shown in *Fig. 2*.

The pathogenicities of the LIVP and LIVPΔati strains were compared at the first stage. The sensitivity of mice to orthopoxviruses depends largely on their age [2]; therefore, young (3-week-old) BALB/c mice were used. The animals (10 mice per group) were i.n. infected with the viruses, since this method mimics the natural route of infection transmission and is responsible for the fact that mice are most sensitive to this very infection [22, 23].

It turned out that upon i.n. infection of young mice, the LIVP strain at a dose of 107 pfu induced the development of a clinically apparent infection (*Fig. 3*) and death of 50% of the animals (*Fig. 4*), while the LIVPΔati strain led to the appearance of less apparent illness signs in mice (*Fig. 3*) and caused the death of 20% of the animals (*Fig. 4*). Hence, the *ati* gene deletion in VACV LIVP caused its attenuation compared to the original viral strain. That is consistent with the previously held assumption that the reduced pathogenicity of some natural VACV strains can be caused by a spontaneous deletion of the *ati *gene in them [13], although experimental evidence to support this has not been provided.

The immunogenicity of VACV LIVP and LIVPΔati was studied in adult mice (aged 6–7 weeks) with a mature immune system. The development of a VACV-specific cell-mediated immune response to i.d. vaccination of mice was assessed by ELISpot, according to the number of splenocytes producing IFN-γ in response to stimulation with peptides. A strong cellmediated immune response was observed already on 7 dpi, reaching a maximum on 14 dpi and significantly declining by 21 dpi (*Fig. 5*). Meanwhile, deletion of the *ati* gene in VACV LIVP affected neither the dynamics nor the strenght of the cell-mediated immune response to vaccination in mice.

The antibody response is known to make the greatest contribution to the eliciting of the adaptive immune response to vaccination with VACV [3, 24]. Therefore, the dynamics of synthesis of IgM and IgG specific to the virion proteins of the VACV after i.d. vaccination of mice with the LIVP or LIVPΔati strain at doses of 105 pfu were assessed by ELISA.

A relatively high IgM level was detected on 7 dpi, attaining its maximum on 14 dpi and then declining. Both VACV strains did not differ in terms of the dynamics and the level of production of virion-specific IgM (*Fig. 6A*).

Intense synthesis of VACV-specific IgG was observed starting on 14 dpi; the antibody level subsequently increased on 21–28 dpi. LIVPΔati was noticeably superior to LIVP in terms of the geometric means of inverse IgG titers on 28, 42, and 56 dpi; this superiority was statistically significant on 42 and 56 dpi (*Fig. 6B*). One of the plausible reasons for this is that the absence of synthesis of the major, late nonstructural protein A29 in LIVPΔati does not distract the immune system from synthesizing IgG specific to this protein and ensures a more intense synthesis of antibodies specific to the virion proteins of VACV.

A number of studies have demonstrated that the humoral immune response makes the greatest contribution to the protection against a challenge with orthopoxviruses [3, 6, 24, 25]; therefore, it was important to assess the protective immunity that had developed in response to i.d. vaccination of mice with VACV LIVP and LIVPΔati. For this purpose, six mice in each group were i.n. infected with lethal doses of CPXV GRI-90 or ECTV K-1 on 60 dpi. In both cases, the protective effect of vaccination with LIVPΔati was stronger than that for vaccination with the parent LIVP strain (*Fig. 7*, *Fig. 8*), *Fig. 9*), which supports the earlier conclusions about the crucial role of the antibody response in the development of a body’s defense against a orthopoxvirus infection.

A conclusion can be drawn that deletion of the genomic region of VACV LIVP comprising the *ati* gene weakens the pathogenic properties of the LIVPΔati virus upon i.n. infection of BALB/c mice and increases the production of virion-specific IgG in response to i.d. vaccination of mice with this mutant virus, thus ensuring stronger protection for mice (compared to the parent LIVP) against the subsequently induced lethal infection with the heterologous orthopoxviruses CPXV and ECTV. Therefore, the LIVPΔati strain can be considered a promising vector for constructing polyvalent recombinant vaccines against various infectious diseases.

## Abbreviations

CPXV: cowpox virus
ECTV: ectromelia virus
VACV: vaccinia virus
pfu: plaque-forming units
dpi: day post immunization
i.d.: intradermal
i.n.: intranasal
